# Natural Language Processing in Diagnostic Texts from Nephropathology

**DOI:** 10.3390/diagnostics12071726

**Published:** 2022-07-15

**Authors:** Maximilian Legnar, Philipp Daumke, Jürgen Hesser, Stefan Porubsky, Zoran Popovic, Jan Niklas Bindzus, Joern-Helge Heinrich Siemoneit, Cleo-Aron Weis

**Affiliations:** 1 Mannheim Institute for Intelligent Systems in Medicine (MIISM), Medical Faculty Mannheim, Heidelberg University, 68167 Mannheim, Germany; 2 Institute of Pathology, Medical Faculty Mannheim, Heidelberg University, 68167 Mannheim, Germany; juergen.hesser@medma.uni-heidelberg.de (J.H.); zoran.popovic@medma.uni-heidelberg.de (Z.P.); jan.bindzus@stud.uni-heidelberg.de (J.N.B.); siemoneit@stud.uni-heidelberg.de (J.-H.H.S.); 3 Averbis GmbH, 79098 Freiburg, Germany; philipp.daumke@averbis.com; 4 Data Analysis and Modeling, MIISM, Medical School, Interdisciplinary Center for Scientific Computing (IWR), Central Institute for Computer Engineering (ZITI), CZS Heidelberg Center for Model-Based AI, Heidelberg University, 69117 Heidelberg, Germany; 5 Institute of Pathology, Medical Faculty Mainz, University Hospital Mainz, 55131 Mainz, Germany; stefan.porubsky@unimedizin-mainz.de; 6 Institute of Pathology, Medical Faculty Heidelberg, 69120 Heidelberg, Germany

**Keywords:** NLP, text analysis, nephropathology, text classification, topic modelling, BERT, transformer encoder, machine learning, deep learning

## Abstract

Introduction: This study investigates whether it is possible to predict a final diagnosis based on a written nephropathological description—as a surrogate for image analysis—using various NLP methods. Methods: For this work, 1107 unlabelled nephropathological reports were included. (i) First, after separating each report into its microscopic description and diagnosis section, the diagnosis sections were clustered unsupervised to less than 20 diagnostic groups using different clustering techniques. (ii) Second, different text classification methods were used to predict the diagnostic group based on the microscopic description section. Results: The best clustering results (i) could be achieved with HDBSCAN, using BoW-based feature extraction methods. Based on keywords, these clusters can be mapped to certain diagnostic groups. A transformer encoder-based approach as well as an SVM worked best regarding diagnosis prediction based on the histomorphological description (ii). Certain diagnosis groups reached F1-scores of up to 0.892 while others achieved weak classification metrics. Conclusion: While textual morphological description alone enables retrieving the correct diagnosis for some entities, it does not work sufficiently for other entities. This is in accordance with a previous image analysis study on glomerular change patterns, where some diagnoses are associated with one pattern, but for others, there exists a complex pattern combination.

## 1. Introduction

Due to complex histomorphological change patterns and diagnoses, nephropathology is a challenging sub-discipline of surgical pathology [1]. This field is hard to learn for beginners, which is reflected, among other things, in a steep learning curve. However, after such a learning process, many assessments by experts in the field show strong inter-observer agreement between results. The high-level specialization of such pathologists is achieved by long, tedious training, which makes them as rare as they are necessary [2]. One idea for assisting novices in the learning process is to utilise machine learning (ML) tools to assist in reaching plausible differential diagnoses or even the correct diagnosis [3,4]. For instance, there were several works from the field of image analysis or respectively computational pathology published by many different groups in recent years [5,6]. In this context, we have also recently published a paper on the classification of glomerular changes in histological images by means of convolutional neural networks (CNNs). Based on a defined small number of change patterns, we were able to diagnose entities defined by only a small number of patterns [7]. For instance, on the basis of images of a patients glomeruli, amyloidosis and diabetic glomerulopathy are easy to predict [1,7,8]. A diagnosis like lupus nephritis, which can show a plethora of patterns over time and space (within one biopsy), is in contrast not predicable solely based on one glomerular change pattern [1,7,8]. Demonstrating that only a part of kidney tissues (in our case glomeruli) is not enough to make a correct diagnosis is not surprising. It seems logical that at least the entire tissue needs to be taken into account; if not, disease models or pathophysiological contexts would have to be included in the diagnostic classification task.

An analysis tool for all kidney tissue compartments, e.g., by combination of a segmentation model to obtain the compartments of interest and subsequent classification, needs to be trained on larger and diverse data sets. Typically, such image data sets are very sparse or, respectively, not easy to create. The problem is not so much the preparation of a compilation of final diagnoses and images, but rather the laborious generation of correct annotations. As an example, in our recent publication, three experts spent several weeks classifying individual images in order to generate a sufficiently large data set [7]. For a segmentation task, where every part of the images needs to be labelled, the effort is significantly higher.

In contrast to this image data scarcity, there is plenty of high-quality text data in the field of nephropathology. For every kidney biopsy, there is a medical report that contains a short description of the histology. These texts are each written by a professionally trained but most likely not-always-available nephropathologist. Furthermore, as mentioned above, for many entities, there is high agreement between these experts. In summary, most image data is not very well annotated; however, the quality of most diagnosis text is presumably very high.

This leads to the idea of using the diagnosis text for analysis, in contrast to our recent work on image data [7]. In a sense, this text analysis is a surrogate for non-existent image data and image analysis tools. Analysing texts instead of images, of course, requires methods of natural language processing (NLP).

Like image analysis, NLP includes a wide range of methods for many different areas of application. In the medical field, the analysis and especially classification of surgical pathology report texts is a well-known application. For instance, there are cancer registries that rely on information extraction from pathology reports or on the classification of such reports. The manual information extraction from (bio-)medical free-text documents and especially pathology reports is very time consuming and requires the commitment of specialists. Automatic, pre-existing **NLP**-approaches provide a solution to overcome this obstacle. For the described cancer registry task, Schulz et al. combined several different classification techniques to extract a particularly large quantity of different information such as cancer type (by e.g., support vector machine (SVM) or tumor morphology (by e.g., convolutional neural network (CNN) with embeddings) from German texts [9]. Besides this mentioned example, there are already numerous other works for the classification of medical texts. Fabacher et al. trained an SVM as a binary text-classfier for French texts [10]. And Oleynik et al. trained an SVM to classify pathology reports according to the International Classification of Diseases for Oncology (ICD-O) code [11,12]. The aim in a recent work by Lopprich et al. [13] was to make a manual documentation process more efficient by using methods of NLP for multiclass classification of diagnostic reports to automatically document the diagnosis and status of disease of myeloma patients.

Against this background, the main aim of this work was to test if the textural description of the entire kidney tissue in (German) nephropathology reports can be used to make a diagnosis or respectively assign the report text to the correct diagnosis. For this purpose, each nephropathological report was divided into two parts, each of which belonged to each other: Part one is the microscopic description section; and part two is the corresponding diagnosis section. As for image data, manual annotation of the cases is sparse. Therefore, we use a two step approach: (i) First, the text-classification task was preceded by a topic modelling task in order to summarize the many given, each individually formulated diagnosis sections into less than 20 diagnosis clusters, where each cluster is a collection of thematically related documents, representing a certain diagnostic group. By doing so, we avoid manual labelling. (ii) Second, different text classification methods were used to predict the corresponding diagnostic group, obtained in step (i), on the basis of the given description section. This tests whether the text description (as a surrogate of the image analysis) contains all the information necessary to generate the correct diagnosis.

For the steps (i) and (ii), different text clustering- and text classification-methods were applied. Overall, we experimented with simple Bag-of-Words (BoW)-based methods (Section 2.3.1 and Section 2.5.1) as well as with techniques based on distributed representations (Section 2.3.2 and Section 2.5.2) to solve the given NLP problems.

## 2. Materials and Methods

### 2.1. Data Collection

Anonymized medical reports (n = 1185 from the years 2018–2021, memory size: 5 MB) were retrieved from the electronic archive of the Institute of Pathology, Medical Faculty Mannheim, Heidelberg University. Only the plain texts are used without information on patient age, gender, clinical course, etc. The data collection and all experiments were conducted in accordance with a vote of the ethics commission II of the Heidelberg University (vote 2020-847R). The total corpus consists of 152,650 words, with each report consisting of 136 words on average.

### 2.2. Overview

**Figure 1:**
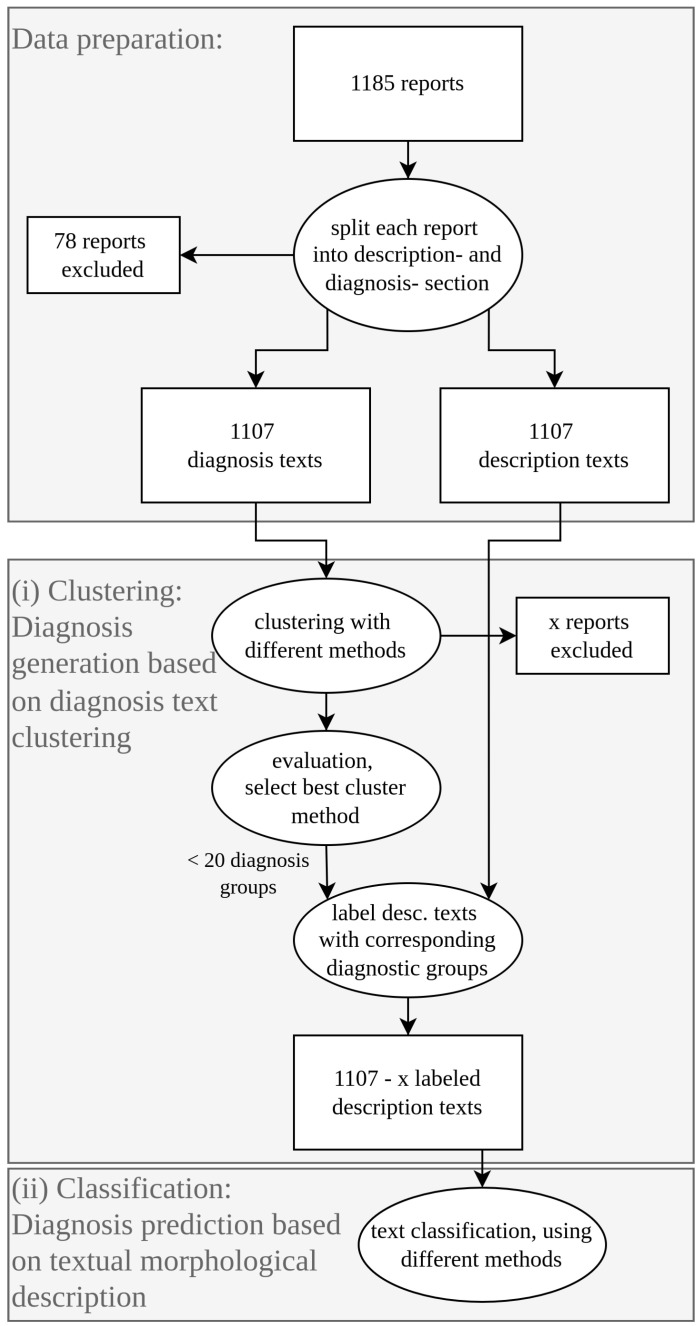
Flowchart, describing the general procedure of the project. After splitting each nephropathological report into its diagnosis and description section (data preparation), we first applied the clustering task (i) to the diagnosis texts in order to summarize them into less than 20 clusters. After labelling each cluster of diagnosis texts with a corresponding diagnostic group, we applied the classification task (ii) to the description texts in order to find out if it’s possible to predict the correct diagnostic group of a given description text with NLP techniques.

An overview of what has been done in the underlying work is provided by Figure 1.

We started with a corpus consisting of 1185 nephropathological reports. Each report was then divided into its diagnosis section and microscopic description section (Figure 1: data preparation). This was done based on German section tags or keywords usually placed at the beginning of a section, like “Klinische Angaben” (Engl. clinical information) for the clinical information section, “Mikroskopie” (Engl. microscopy) for the description section, and “Beurteilung” (Engl. conclusion) for the diagnosis section. The diagnosis section is later used for the text clustering task (i), and the description section is later used for the text classification task (ii).

Below is an example of a conclusion text with its associated microscopic description section:

  Example of a microscopic description section (translated from German to English):

*Renal medulla and cortex with 18 glomeruli. These were inconspicuous by light microscopy, specifically without evidence of necrosis or extracapillary proliferation. Arcuate artery and interlobular artery with mild subendothelial fibrosis. Arterioles unremarkable.*

*Tubulointerstitium with only small areas of atrophic tubules and interstitial matrix proliferation. Percentage of chronically damaged tubulointerstitium: 5%.*

  Example of corresponding diagnosis section (translated from German to English):

*Mild arteriosclerosis. Unremarkable chronic tubulointerstitial damage (5% of the cortex). Conventional microscopy moreover an unremarkable finding with no evidence of glomerular necrosis or extracapillary proliferation. The results of the further immunohistochemical examination will be reported afterwards.*

**UMLS! (UMLS!)** [14] was used for the translation (German to English) in order to use internationally standardized medical terms if possible.

Some reports (78) did not meet all requirements and could therefore not be divided into the two sections and were excluded. After that, 1107 reports are left, consisting of one diagnosis and one description text.

After data preparation, two main tasks were performed: In the *clustering task (i)*, diagnoses were assigned to the description texts. In the second step, the *classification task (ii)*, the aim was to predict the correct diagnosis for each (morphological) description text, using different text classification methods.

Here are more detailed descriptions of the two main tasks:

(i) *Clustering and topic modelling*
  The diagnostic segments (example above) are clustered using different approaches (as described below in Section 2.3). The results of the various clustering methods are then compared to select the best method, which can be used for the topic modelling task of the corpus. The clusters of the winner are then analyzed in more detail to assign a suitable diagnostic group name for each cluster. After that, we obtained a labelled corpus, where each report is labelled with one diagnostic group that can be identified using the associated cluster index.
(ii) *Classification*
  The classification part (Section 2.5) involves testing how accurately the labelled microscopic description texts can be classified using different text classification methods (as described below in Section 2.5). The aim is to find out whether the descriptive texts contain enough information to predict the diagnosis. For the classification task, different text classification methods were tested, to compare how they differ from each other in terms of performance.

All text processings, analyses, and evaluations performed in this thesis were conducted with German texts. In principle, the analyses shown here can be applied to reports written in any other language. More about this is mentioned in Section 4.3. Furthermore, only freely available software was used in this work. All python libraries used are referenced in the appropriate places (via hyperlink and or citation). The code for this work is available on GitLab (see section *Data Availability Statement*).

### 2.3. Clustering and Topic Modeling

We tested and compared seven different clustering approaches to cluster the diagnosis sections of the given reports. The resulting clusters were then used as labels for the classification task (ii). Here, a trade-off was necessary between too few clusters (or respectively labels or diagnostic groups) having a high intra-cluster heterogeneity and too many clusters with low intra-cluster heterogeneity but only a few cases. Too few clusters would generally be easier for a prediction model. With too many, on the other hand, the low number of cases per group would be problematic. To balance this, the amount of clusters was set to a minimum of 10 and a maximum of 20.

The used clustering methods can be divided into two main categories: BoW-based approaches (Section 2.3.1) and approaches with distributed representations (Section 2.3.2), where we make use of word embeddings and pre-trained transformer encoder models.

#### 2.3.1. Clustering with Bag-of-Words Approaches

The clustering methods used in the underlying work, which are based on BoW representations [15], are listed here:

- *k-means*
  K-means clustering, using scikit-learn’s [16] python implementation.  
- *LDA*
  **LDA! (LDA!)** clustering, using the implementation shown in [17]  
- *HDBSCAN*
  **HDBSCAN! (HDBSCAN!)**, as shown in [18], using the supplied python library hdbscan. Before applying HDBSCAN, we first reduced the dimensionality of the document vectors as the HDBSCAN clustering algorithm handles high dimensionality poorly. We used Uniform Manifold Approximation and Projection (UMAP) [19] for the dimensionality reduction.  
- *GSDPMM*
  **GSDPMM! (GSDPMM!)** model for text clustering [20].  

For text vectorization, term frequency–inverse document frequency (tf–idf) has been used (using scikit learn’s implementation). Moreover, the text has been pre-processed intensively to keep the vocabulary small, which results in smaller document vectors. We used stop word filtering, with general purpose German stop words, using the nltk.corpus [16] package (slightly customized by removing words like “no” or “none” from the predefined stop words list and adding words like “approx”), as well as lemmatization with the (German) Hanover Tagger [21]. We expanded this lemmatizer with custom word replacements, to adapt it to our specific nephropathological language. Moreover, we used a multi-word expression tokenizer (nltk.tokenize.mwe), to merge multi-word expressions, like “Lupus␣Nephritis” (Engl. lupus nephritis) or “tubulointerstitieller␣Schaden” (Engl. tubulo-interstitial damage), into single tokens. We used an elbow-method-based approach to find the optimal number of clusters (*k*) for each cluster method. We removed numbers like dates, quantities, or report identification numbers to prevent reports from being clustered only by irrelevant numerical values. Furthermore, we used uncased texts, and we removed punctuation.

#### 2.3.2. Clustering with Distributed Representations

Distributed representations of documents and words led to a considerable breakthrough in NLP due to their ability to capture the semantics of words or even word sequences. Word embeddings, or contextual word embeddings from transformer encoder models, can provide a certain language or textual context understanding, which is required for many NLP tasks and is also useful for clustering and topic-modelling problems [22,23,24].

- *top2vec*
  top2vec [24], uses distributed representations, obtained with word2vec [25] and doc2vec [26], to measure the semantic similarity of documents.  
- *BERT-based clustering*
  Since the break through of the Bidirectional Encoder Representations from Transformers (BERT) model [27], a huge collection of pre-trained transformer encoder models have become available for various domains. Most of them are freely available on platforms such as huggingface.co [28]. We used different pre-trained transformer encoder models to embed the diagnostic texts in 512-dimensional document vectors (as shown in [29], using the supplied sentence_transformers library). After reducing the dimensionality of the document vectors (bidirectional, contextual embeddings) with UMAP [19], we clustered the documents using **HDBSCAN!** [18].
  The following BERT-based models were used in this work:  
  - *German-BERT*
    There are some promising pre-trained transformer encoder models for the bio-medical domain [30,31,32], but these models have only been trained with English texts. Since we are dealing with German bio-medical language, we used bert-base-german-cased (henceforth called *German-BERT*) which has been pre-trained with German wikipedia articles, the *OpenLegalData* dump and news articles. German-BERT comes with BERTs *WordPiece tokenizer* [27] (30,000 token vocabulary) which is able to divide unknown words into known subwords. Therefore it can be used for a wide range of domain-specific languages without getting many **OOV!** (**OOV!**) cases. Only one OOV case appeared during the tokenization of the entire corpus with German-BERT with **OOV!** token “*q:*”.
  - *Patho-BERT*
    In order to adapt German-BERT to our specific nephropathological vocabulary, we pre-trained it with a masked language modelling) (MLM) objective, using the whole nephropathological corpus and 1607 additional nephropathological reports as training data. The resulting model was then saved as ger-patho-bert (henceforth called *Patho-BERT*) and used as another transformer model for further clustering attempts as well as for classification tasks in Section 2.5.

When working with distributed representations, little to no text pre-processing is usually required [33]. However, irrelevant numbers have been filtered out to prevent clustering based on numerical values only, as explained in Section 2.3.1.

### 2.4. Evaluation of Clustering Results

Each clustering method divides the included 1107 diagnosis texts into less than 20 clusters. In other words, each clustering method generates a set of clusters, henceforth called *cluster-set*. Now the question arises how we can evaluate the quality of such a cluster-set. For this we have taken into account the shape of the cluster-set as well as its texts contained in each cluster in order to find the most homogeneous and diagnostically meaningful clusters.

#### 2.4.1. Clustering Metrics

There is still no perfect standard way to evaluate the quality of a cluster-set. In some publications, metrics like purity or **NMI! (NMI!)** are used to evaluate and compare clustering results [20,34,35,36]. However, a so-called *golden cluster-set* is required for such metrics, which is not available in our case. The present work generated clusters using different methods without ground truth data. This significantly limits the number of cluster metrics that can be used. The following methods were used to measure the overall clustering quality:

- *silhouette score*
  The mean silhouette coefficient of all samples, using scikit-learn’s implementation. This metric is generally higher for convex clusters and is therefore not suitable for every cluster-set.  
- *relative entropy*
  The entropy of the documents, relative to the clusters. It is a measure of how much the documents differ from all other documents in the same cluster (regarding term frequency). A small value means that the documents of a cluster are similar in terms of vocabulary (on average). We calculated the relative document entropy as follows:
  $${\mathrm{mean}}_{j=1}^{m}\left({\mathrm{mean}}_{i=1}^{n}\left(\mathrm{entropy}\left(\mathrm{tf}\left({\mathrm{doc}}_{i,j}\right),\mathrm{tf}\left({\mathrm{cluster}}_{j}\right)\right)\right)\right)$$
  where tf is the term frequency (calculated with scikit learn’s CountVectorizer) and $\mathrm{entropy}(\mathrm{tf}\left({\mathrm{doc}}_{i,j}\right),\mathrm{tf}\left({\mathrm{cluster}}_{j}\right))$ is the entropy of the *i*-th document of cluster *j*, relative to all other documents of cluster *j*. The entropy was calculated with scipy’s entropy function, which uses the Kullback–Leibler divergence.  
- *classification accuracy (cls accuracy)*
  The idea is to test how well a simple **SVM!** (**SVM!**) can classify a given cluster-set, as it was also done in [35] to compare different topic models. The diagnosis sections of the reports are the input of the SVM and the labels to be guessed are the corresponding clusters.

#### 2.4.2. Visual Presentation of Clustering Results

We visualized the data points of each cluster-set with **UMAP!** (**UMAP!**) [19] in order to get an impression of how well the clusters are separated from each other.

#### 2.4.3. Keywords Extraction

In order to determine the topics of the individual clusters, the most relevant words (henceforth called *keywords*, or *topic words*) have to be extracted from the clusters. Two methodologically different approaches were used for this purpose. First, term frequency–inverse document frequency (tf–idf) as term frequency-based method was used. Here, keywords are identified based on their different frequencies in the clusters. Second, we used an “***SVM!**-based*” topic words extraction method, which is based on the model explainability of an **SVM!**. After training a linear SVM to predict the clusters of each diagnosis text, we applied a weight analysis to the SVM, in order to get the ten words which make the SVM most likely to predict a particular cluster (using the eli5 module). Only the documents predicted correctly by the SVM were included in the analysis.

#### 2.4.4. Cluster Naming Based on Keywords

After keyword extraction, medical experts (JNB and CAW) then mapped proper diagnostic group names for each cluster (see Section 3.1.2).

### 2.5. Classification

In the previous section, we clustered the diagnosis sections of the reports into different cluster-sets with different clustering methods (bag of word-based and embedding-based). The hypothesis is that each cluster represents a diagnostic group (e.g., Lupus nephritis), which should hypothetically result from the associated microscopic description section of the same report.

To test this hypothesis, we tested how well these microscopic descriptions can be classified to the corresponding diagnostic group (represented by the clusters of a given cluster-set), with machine learning techniques. Therefore, we trained and tested different text classifiers which are typically used in **NLP! (NLP!)**. These again include simple **BoW! (BoW!)**-based methods (Section 2.5.1) as well as more advanced techniques, based on embeddings and transformer encoder models (Section 2.5.2).

#### 2.5.1. Classification with Bag-of-Words Approaches

First, the description texts are pre-processed and tokenized with the same techniques, as for the **BoW!**-based clustering in Section 2.3.1. The tf–idf-vectorized description-texts are then passed to one of four different classifiers for the final prediction:

- *SGD-classifier*
  **SVM! (SVM!)** with SGD learning.  
- *MLP-lassifier*
  **MLP! (MLP!)** classifier with Adam optimization.  
- *Logistic Regression*
  Logistic regression (aka logit, MaxEnt) classifier with regularization and multinomial loss fit.  
- *Multinomial NB*
  Multinomial **NB! (NB!)** classifier.

All **BoW!**-based classifiers are implemented with scikit-learn [16].

#### 2.5.2. Classification with Distributed Representations

In addition to **BoW!**-based classification, we also made use of classification methods, which are based on distributed representations. Bidirectional recurrent neural networks and convolutional neural networks with word embeddings, as well as BERT-based transformer encoder models were tested:

- *RNN + embeddings:*
  **RNN! (RNN!)**, consisting of a bidirectional **LSTM!** (**LSTM!**) layer, trained together with word2vec word embeddings as input.  
- *CNN + embeddings:*
  **CNN! (CNN!)**, trained together with word2vec word embeddings as input, as shown in [37]. The 1D convolution layer has been trained with 32 kernels with a size of 3, followed by a max pooling layer and two fully connected layers to get one final prediction value for each class. We used the **ReLU! (ReLU!)** activation function for the convolution layer, as well as for the first dense layer. For the last dense layer, we used a *softmax* activation function.  
- *German-BERT:*
  The transformer model bert-base-german-cased, fine-tuned with our text classification problem.
- *Patho-BERT:*
  Our pre-trained Patho-BERT transformer, as introduced in Section 2.3.2.

Both, the **RNN!**- and the **CNN!**-approaches are implemented with tensorflow [38]. We used the transformers package from huggingface [28] for the implementation of all transformer-based methods and trained the models with the included pytorch [39] Trainer API, which uses an adam optimizer with weight decay regularization as introduced in [40].

The texts were pre-processed using the same techniques, as mentioned in Section 2.3.2.

### 2.6. Evaluation of Classification Results

To evaluate and compare different classifiers with one another, we measured various metrics such as accuracy, precision, recall, F1-score (the harmonic mean of precision and recall), and the cohen’s kappa coefficient [41]. Each metric value was determined using ten-fold cross-validation. In order to examine the classification ability of a classifier in more detail, confusion matrices were plotted and analyzed.

## 3. Results

### 3.1. Topic Moelling Based on Text Clustering on the Diagnosis Section of Nephropathological Reports (Ad Task I)

Before documents can be classified, the number of possible classes should be reduced. To accomplish this, the text-classification task was preceded by a topic modelling task (task i). This was done by testing different text-clustering approaches to find the one resulting in the most homogeneous and diagnostically meaningful clusters.

#### 3.1.1. What Are the Differences of the Tested Clustering Methods?

**Figure 2:**
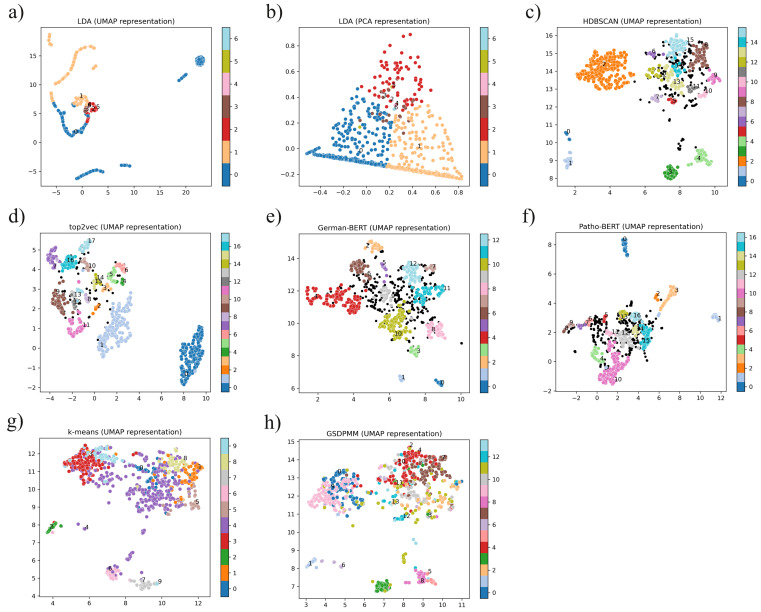
**UMAP!** (**UMAP!**) and **PCA!** (**PCA!**) of different cluster-sets. UMAP representations of the cluster-sets generated with (**a**) **LDA!** (**LDA!**), (**c**) **HDBSCAN!** (**HDBSCAN!**), (**d**) top2vec, (**e**) German-BERT, (**f**) Patho-BERT, (**g**) k-means and (**h**) **GSDPMM!** (**GSDPMM!**). The **LDA!** cluster-set is also shown as **PCA!** (**PCA!**) in (**b**). Each data point represents a diagnosis section of a report. The data points are coloured according to the respective clusters. Black points represent outliers that were not assigned to any cluster. Above all, the clusters of top2vec and **HDBSCAN!** appear particularly tidy and separated. The clusters of k-means and **GSDPMM!** appear less well separated, which is probably also due to the fact that no data points are sorted out here.

In the present work, **BoW!**-based approaches and embedding-based approaches were used to cluster the given diagnosis texts into several diagnostic groups. As a metric for the clustering quality, we used the **s-score!** (**s-score!**), relative entropy, and the classification accuracy. The silhouette-score assumes convex cluster shapes and is therefore not well-suited for clusters of other shapes. To be independent of the cluster shape, the **SVM!**-classification-based **cls accuracy!** (**cls accuracy!**) (as described in Section 2.4 above) is used as additional clustering metric. Table 1 shows the measured metric values for each clustering approach and Figure 2 shows the UMAP-representations of the respective cluster-sets.

Compared by visual inspection to all other tested clustering methods, the clusters of k-means (Figure 2g) and **GSDPMM!** (Figure 2h) seem to be much more poorly separated, which is also reflected in their low silhouette scores in Table 1. Interestingly, **HDBSCAN!**, a **BoW!**-approach, achieved the highest silhouette-score, the highest cls accuracy and the second best entropy value. Moreover, it turned out that a reasonably shaped cluster-set is not necessarily easier to predict with a support vector machine: Although top2vec has achieved a good silhouette-score (s-score: 0.545) and shows well separated clusters in its **UMAP!**-representation (Figure 2d), an **SVM!** can’t predict the clusters very well (cls accuracy: 0.372). Top2vec has with 0.780 the highest relative entropy value, which hints to a low intra-cluster heterogeneity. This heterogeneity could be one reason why top2vec-clusters are so difficult to predict. On the other hand, k-means and GSDPMM achieved the lowest silhouette-scores, but are quite well predictable with a cls-accuracy of 0.905 (k-means) and respectively of 0.805 (GSDPMM). Both methods also have lower entropy values with 0.612 and respectively 0.675 than top2vec.

For LDA, k-means and GSDPMM, no outlier detection has been implemented. Contrary, outliers can be detected for the other clustering techniques and subsequently be removed from the further analysis. Especially in the case of Patho-BERT and German-BERT, several documents were identified as outliers, which reduced the amount of left documents-the *corpus size*-noticeably from 1107 documents to less than 760.

A fairly imbalanced cluster distribution can be found in almost every cluster-set. However, such uneven distributions of cases is nothing unusual in this domain, as some diseases occur much less frequently than others.

#### 3.1.2. Can the Clusters Be Named on Basis of Keywords?

All cluster metrics used so far have the disadvantage that one cannot derive diagnostically comprehensible clusters from them. These methods only give a metric for the intra-cluster homogeneity.

To give the produced clusters meaningful names, we first used different keyword extraction methods (described in Section 2.4.3 above). The extracted topic words of the HDBSCAN cluster-set (translated from German to English) can be found in Table 2 and Table 3. The original (German) topic word tables can be found in Appendix A and the extracted topic words of all other cluster-sets can be found at https://doi.org/10.11588/data/KS5W0H (accessed date 14 July 2022). As explained in Section 2.4, the ten (by each keyword extraction approach identified) most relevant words per cluster are shown in these topic word tables.

Second, two medical experts (JNB as medical student and CAW as board-examined pathologist) analyzed these tables to find out how well the topic words of each cluster fit together and whether the topic words of a cluster fit to a certain diagnostic or topic group (henceforth called *cluster name*).

The medical experts annotated the topic word tables as follows: If a suitable name was found for a cluster, the cluster name can be found next to the corresponding cluster index (see Table 2, left column). A particularly large number of topic words strongly refer to cluster names highlighted in green (*strong cluster names*). In the case of cluster names marked in orange, only a few topic words indicated the specified cluster name (*weak cluster name*). The same applies to the colour-coded topic words: topic words that strongly indicate a cluster name are highlighted in green (*strong topic words*). Orange highlighted topic words only weakly indicate a cluster name (*weak topic words*).

Especially in the case of **HDBSCAN!**, for many clusters the extracted keywords fit thematically well together. In this case, diagnostically meaningful and comprehensible cluster names based on the keywords could be assigned to 14 out of 16 detected clusters (as shown in Table 2 and Table 3). For instance, keywords like “lupus nephritis” or “chronicity index” are characteristic for documents in the cluster “systemic lupus erythematosus”. Or for “IgA-nephritis” words like “oxford” or “IgA” are typical.

#### 3.1.3. Do the Authors of the Nephropathological Reports Have an Impact on the Clustering?

With only three authors (CAW, SP and ZVP) writing in different combinations the included reports, we wondered if the clustering is influenced by the different authors. It is conceivable, for example, that one of the authors is an expert in a particular diagnosis and at the same time has a characteristic wording. In this case, the clustering methods would possibly be influenced by the wording.

**Figure A1:**
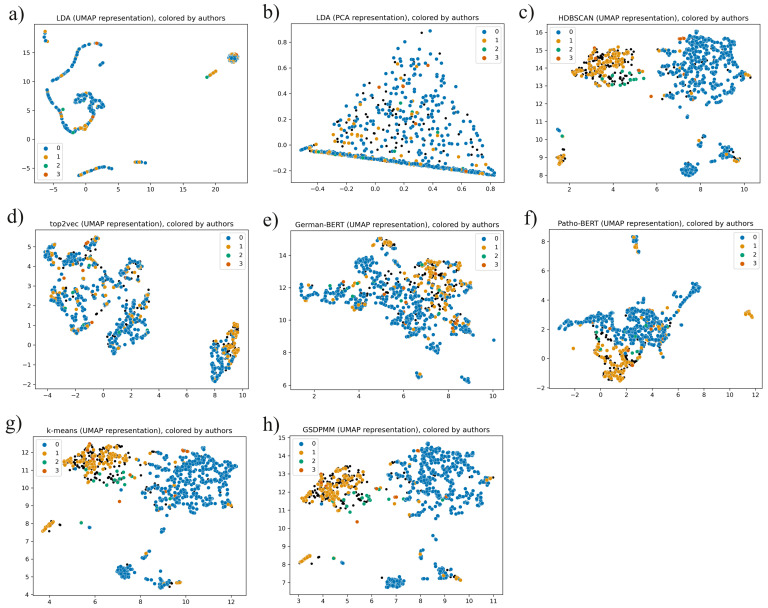
**UMAP!** (**UMAP!**) of the cluster-sets generated with (**a**) **LDA!** (**LDA!**), (**c**) **HDBSCAN!** (**HDBSCAN!**), (**d**) top2vec, (**e**) German-BERT, (**f**) Patho-BERT, (**g**) k-means and (**h**) **GSDPMM!** (**GSDPMM!**). The **LDA!** (**LDA!**) cluster-set is also shown as **PCA!** (**PCA!**) in (**b**). Each dot colour represents a different author. The authors of the reports marked in black are unknown (e.g. because multiple authors were involved).

To look for the authors, Figure A1 (see Appendix B) shows the same **UMAP!** plots as Figure 2, but coloured according to the authors who wrote the respective reports. Some of the reports were written by multiple authors, these are represented by black points. When examining these figures, especially **HDBSCAN!**, k-means and **GSDPMM!** tend to form a group for author 1 (orange dots) and author 2 (green dots) that is separate from author 0 (blue dots). This group is always located in the upper left area. Based on this, the question arises whether these separations are due to the different writing styles of some authors or whether the authors worked on different subject areas. In the manual cluster word analysis, some clusters could be identified, which were probably mainly grouped according to the language style of author 1, e.g., cluster 2 of the HDBSCAN cluster-set. The topic words of this cluster refer only weakly to the topic *tubulo-interstitial nephritis* (see Table 2 and Table 3).

#### 3.1.4. Which Clustering Method Is the Best?

Since the **HDBSCAN!**-clustered data set has a good clustering accuracy according to the applied metrics (compare Section 2.4), since it has less outliers than German-BERT or Patho-BERT, and, since it achieved decent results in the manual topic word analysis (compare Section 2.4.3), it has been rated as the best clustering approach. Therefore, it, or rather its cluster-set, has been used as the target for the classification task described in the next section.

### 3.2. Assignment of Nephropathological Description Sections to Specific Diagnostic Groups (Ad Task Ii)

The second task is mapping the histomorphological descriptions to the diagnosis sections or, more specifically, to the previously defined topics (task ii). Based on Section 3.1.1, **HDBSCAN!** was selected as the preferred clustering method on which basis different classifications methods were tested.

#### 3.2.1. Which Methods Can Be Used to Map Descriptive Sections to the Correct Diagnostic Groups?

In summary, eight different text classification methods were used. Four of the classification methods (here abbreviated called SGD-classifier, **MLP!**-classifier, logistic regression and multinomial **NB!**) are bag-of-words approaches (compare Section 2.5.1). The other four classification methods (here abbreviated as RNN + embeddings, CNN + embeddings, German-BERT and Patho-BERT) are in contrast based on distributed representations (compare Section 2.3.2).

In Table 4 the performance of the different approaches for mapping the description sections to the HDBSCAN clustered data set is shown. The performance is quantified by calculating the F1-score and the Cohen’s kappa coefficient with ten-fold cross-validation, as mentioned in Section 2.6.

Interestingly, according to the different metrics used, there is no clear winner when comparing embedding-based approaches to **BoW!**-based approaches. There are poorly-performing and better-performing models on both sides. Patho-BERT performed higher (best F1-score and Cohen’s kappa coefficient) compared to the other classifiers. So the time-consuming pre-training of a BERT model with **MLM! (MLM!)** seems to be worthwhile in this case. But surprisingly, the SGD-classifier, a BoW-based classifier, achieved a significantly better F1-score than German-BERT. In return, German-BERT achieved a better Cohen’s kappa coefficient.

#### 3.2.2. Can Certain Diagnoses Be Better Predicted than Others? And If So, What Are the Reasons?

**Figure 3:**
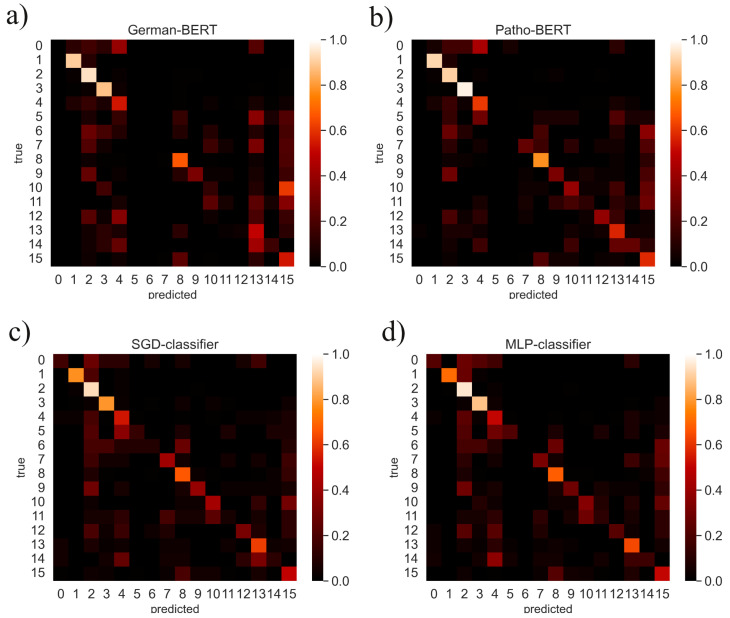
Confusion matrices of the classification models. (**a**) German-BERT, (**b**) Patho-BERT, (**c**) the **SVM!** (**SVM!**)-based SGD-classifier, and (**d**) the **MLP! (MLP!)**-classifier. The brightness of a cell indicates how many times the class on the x-axis was predicted by the classifier. The true class is indicated by the index of the y-axis. Interestingly, there are classes that could be recognized well by all classifiers, including the weaker ones, e.g., class 1 (*rapid progressive glomerulonephritis*), 2 (*tubulo-interstitial nephritis*) and 3 (*pauci immune glomerulonephritis*). Although the transformer-based classifiers (**a**,**b**) generally performed better, the **BoW!**-based methods were able to detect class 0 (*systemic lupus erythematosus*) or 5 (*fsgn*) better (**c**,**d**).

Besides the overall classification performance, as shown above, the performance with view to the single classes was of interest. To visualize this, in Figure 3 the confusion matrices of the four best classification methods are shown. In addition, the F1-scores per cluster can be read in Table 5, which were achieved using Patho-BERT as classifier.

First, it can be observed that the **HDBSCAN!** cluster-set contains some clusters that can be recognized well by all classifiers, even the weaker ones. Especially the clusters 1 (*systemic lupus erythematosus*), 2 (*tubulo-interstitial nephritis*), and 3 (*pauci immune glomerulonephritis*) could be recognized well by all classifiers, according to the confusion matrices (Figure 3). The Patho-BERT model was able to achieve F1-scores of over 0.8 for these three diagnostic groups, with group 3 (*pauci immune glomerulonephritis*) performing best with an F1-score of 0.892 (see Table 5). The clusters 4 (*iga nephritis*), 8 (*kidney transplant*), 13 (*diabetic glomerulosclerosis*) and 15 (*tubulo-interstitial nephritis*) could be recognized moderately good, with F1-scores of over 0.5. Interestingly, although Patho-BERT has the best overall F1-score (Table 1), the **BoW!**-based methods were able to detect class 0 (*systemic lupus erythematosus*) and 5 (*FSGN*) better, according to the confusion matrices (Figure 3). Table 5 also lists the *support* of each cluster. The support indicates how many documents (microscopic description texts) were available for a cluster (or a diagnostic group). Cluster 2 in particular is significantly more supported than all other clusters, while there are some particularly small clusters consisting of less than 20 documents. Such imbalanced datasets can lead classification algorithms to ignore the minority class entirely, as seems to be the case for clusters 0, 5, and 6 in Table 5. Table 5 shows clearly that the lower the support, the more difficult it is to recognize the cluster. Presumably, these diagnostic groups could have been recognized better if more training data had been available or if each cluster had been large enough, respectively.

#### 3.2.3. Which Classification Approach Is the Best?

Overall, the best classification results were obtained using our custom BERT model (Patho-BERT) as well as using a simple **SVM!** (SGD-classifier). However, the description sections of the **HDBSCAN!**-clustered reports could not be classified passably: An F1-score of more than 0.7 could not be achieved, even with our custom pre-trained **BERT! (BERT!)** model (Patho-BERT). In many other medical text classification problems, significantly higher F1-scores could be achieved [9,11,13]. Nevertheless, the problem presented here can hardly be compared with such classification problems, due to the fact that no human-labelled data was available. Instead, we clustered the diagnosis sections to label the data. During development it could be observed that the classification performance was strongly related to the quality of the cluster-set. Moreover, not much training data was available, which resulted in several small clusters consisting of only 10 to 20 documents. In particular, predicting small clusters was hard to accomplish in most cases. Nevertheless, certain clusters are well distinguishable, as mentioned earlier.

## 4. Discussion

Digital medical reports can be found in many different medical sub-disciplines. They usually represent a condensate of one or a combination of the many different, complex, available medical data types such as radiological images, molecular profiles, clinical examination findings, etc. On the one hand, it is of great research interest to obtain usable information for further analyses from medical reports, which are often written in a non-standardized way. On the other hand, the relationship between the underlying data (e.g., histological images) and the text is also of great interest. Against this background, we examined diagnostic texts from the field of nephropathology by means of natural language processing (**NLP!**). In this sub-field of pathology, among others, we were able to show in a recent publication that images of glomeruli can be mapped by means of machine learning to some diagnoses (such as amyloidosis), whereas for other diagnoses (such as lupus nephritis) prediction based on glomerular changes alone does not work well. To test without extensive image processing efforts, if the morphological information in the entire kidney tissue is enough for diagnosis prediction, we examined nephropathological reports. By doing so, we were able to show the following points: (i) First, we could show that long-known **NLP!**-tools like bag-of-word-based techniques and newer embedding techniques likes BERT can be applied to different parts of histological reports written in German (see Section 4.1 below). (ii) Second, we could demonstrate that different text parts like the description or the diagnosis section can be clustered without supervision to diagnostic groups (see Section 3.1.1 above and Section 4.1 below). In contrast to the unsupervised clustering of images, this is much easier and numerous methods are known here from various other fields (see Section 4.1 below). (iii) Third, we could show that these diagnostic groups can be predicted by machine learning models based on the description section (see Section 3.2 above and Section 4.1 below).

### 4.1. Natural Language Processing and Image Processing Techniques in Nephrology and Nephropathology

In this work, we applied a wide range of different **NLP!**-techniques to the histological reports from the field of nephropathology. These medical reports are composed of several sections, with the descriptive and diagnostic sections being particularly relevant for us. We can show that there is a correlation between the morphological description and the final diagnosis by predicting the diagnosis on basis of the description with our custom Patho-BERT transformer encoder model or even with less complex support vector machines (Section 3.2). After a previous work on glomerular change patterns in histological images [7], we used the morphological description by nephropathological experts as surrogate for image analysis. This would have the advantage of eliminating the need to establish a reliable image analysis. For such image analysis, the amount of large, properly annotated datasets is a common bottleneck that we tried to avoid by using textual data [42,43,44].

Of course, textual data must also be prepared or annotated for analysis. We have reduced this workload here in part by using unsupervised clustering methods Section 3.1.1. These clusters or diagnostic groups can then be predicted by a classifier like a support vector machine or with a domain specific pre-trained **BERT!**-based model. Furthermore, by examining the keywords relevant for the respective clusters, one can establish a relationship to diagnostic groups and give the clusters umbrella names such as those in standard nephropathology textbooks [1,8]. The establishment of a relationship between the results of an unsupervised approach and the real-world labels is also a common issue in image analysis. There, the main solution is also to have names assigned to the labels by human experts. For other, also machine-learning-based approaches, such as extending models with a re-mapping block, we were recently unable to show any benefit for lung carcinoma [45].

Our previous focus on image analysis fits quite well into the overall context. In nephropathology, machine learning seems to be mainly used in the form of image analysis [3,4,46,47], but rarely in the form of **NLP!**, although **NLP!** is recognized as a topic of interest [48,49]. This is indeed surprising, since nephropathology seems to be predestined for text analysis due to various standardization efforts. There are, for instance, well written and extensive recommendations on how to write and structure a report [50,51,52,53]; albeit somehow controversial and not followed by everyone. There are also efforts on creating common ontologies, taxonomies, or at least vocabularies for nephropathology [53,54]. The clustering of diagnosis texts or the reduction of different diagnoses to diagnostic groups, which we show here (Figure 2), can be seen in the context of stratification procedures. Since we have not investigated the relationships between clusters here (no ontological approach), nor have we investigated hierarchical relationships (no taxomic approach), our work can be seen as the automatic generation of a vocabulary. In the works dealing with stratification or unification approaches, common vocabularies are described as the basis for more complex tasks like creating a taxonomy or ontology [53,54].

### 4.2. How Do Our Results Fit into the Big Picture?

Even though the method of the present work differs significantly from our previous work, and even though, in contrast to our previous work, the entire kidney tissue or its descriptor was included in the study, the results fit together surprisingly well [7]. Again, certain diagnoses or groups of diagnoses can be predicted very well. For example, amyloid-deposition-associated diseases are again among the best predicted diagnoses. This is not very surprising, since in images it is characterized by typical, amorphous deposits, and in texts it is characterized by the word “amyloid”. In the same way, the IgA nephropathies, for example, are characterized by the description of the typical finding of granular IgA deposits in the mesangium. Since in the previous work only glomeruli without additional staining were analyzed, text analysis is significantly better for diagnoses that are defined by certain, specific findings.

Nevertheless, in several cases the morphological description is apparently not sufficient to make accurate predictions. The combination of image analysis and text analysis as well as the additional integration of patient data or other clinical features could help to identify more correlations and improve the prediction accuracy. Moreover, such diagnosis prediction models could also be used to select and revise potentially incorrect diagnoses.

### 4.3. Other Languages

All text analyses shown in this work have been applied to German reports only. In principle, all methods shown can be applied to documents written in other languages without much additional effort. For the shown **BoW!**-based analyses, some (keyword-based) text pre-processing steps would have to be adapted to the used language. For example, a different stop word list, as well as a different lemmatizer would have to be used. Especially for all transformer encoder based approaches (BERT-classification and BERT-clustering), other pre-trained transformer models would have to be used. Depending on the language, more or less suitable models are freely available. For example, there are many large transformer models for the English medical domain [30,31,32].

### 4.4. Technical Weaknesses and Possible Improvements

One drawback of this work is that the cluster naming (Section 3.1.2) could almost not be evaluated and refined. For this, not enough experts were available. Moreover, only experts trained by the same instructors were available. Therefore, no detailed inter-observer variability studies could be performed to measure the reliability of the cluster topics. For this we would need to recruit more experts from different institutions for future projects.

Moreover, the examined dataset was unfortunately too small to find enough text data for each diagnosis group. This resulted in some particularly small clusters that could hardly or not at all be predicted by any of the tested classification approaches. Resampling-based solutions for imbalanced data (e.g., **SMOTE! (SMOTE!)** [55]) could not be successfully implemented in this work because of the text complexity and the different text vectorization methods used. Another possibility to improve the classification results could be the use of optimization methods for imbalanced classification problems, such as using the dice loss as done in [56]. However, it is also questionable whether this would be effective for the smallest clusters.

In this work we used German-BERT’s word-piece tokenizer for our BERT-based models, since it fits well for German languages and is able to divide unknown medical terms into several known subwords, resulting in very less **OOV!** cases. Although this worked out in principle, using a custom tokenizer, which is specialized to the German nephropathological vocabulary might produce even better classification and or clustering results.

## 5. Conclusions

Overall, it can be said that the morphological description texts, as surrogate for image analysis, enable the correct diagnosis to be achieved for some entities. For other entities, this associative approach does not work adequately. As in our previous image analysis-based study on glomerular change patterns [7], it can be said here that some diagnoses are associated with one pattern, and for others, there is a complex pattern combination which makes the prediction difficult without patho-physiological knowledge. This raises the consideration of including disease models in the analysis to improve accuracy. However, methods such as semantic graphs should perhaps be tested beforehand, as they are much easier to implement.

Besides the only associative approach here, one major issue of this work was the inadequate amount of labelled training data, which is why we performed a time-consuming topic-modelling task first. In general, with more, manually-labelled, balanced data, better text classification results could have been possible. In addition, the classification performance depends on the properties of the given data and on the text pre-processing methods used. These influences were not examined in detail.

The combination of text-based and image-based analysis could be worthwhile in order to be able to take into account additional features regarding the whole tissue in addition to the glomerular changes, which is mainly extracted in the image analysis.

The use of ***VL-PTMs! (VL-PTMs!)*** [57], e.g., *ViLBERT* [58] or *Unicoder-VL* [59], could be a good opportunity to combine image analysis with text analysis in nephropathology. Therefore, sufficient image-text data pairs would be needed. The benefit would be that time-consuming image-labelling would not be necessary.

## Figures and Tables

**Table 1:** Metrics of different cluster-sets.

Cluster Method	s-Score	cls Accuracy	rel Entropy	Clusters	Corpus Size
HDBSCAN	0.587	0.951	0.588	16	906
German-BERT	0.576	0.856	0.618	13	759
top2vec	0.545	0.372	0.780	18	1026
Patho-BERT	0.536	0.848	0.531	17	757
LDA	0.517	0.581	0.611	7	1107
k-means	0.038	0.905	0.612	10	1107
GSDPMM	0.033	0.805	0.675	14	1107

We used the silhouette score (s-score), relative entropy (rel entropy) and the **SVM! (SVM!)**-based classification performance (cls accuracy) to evaluate and compare different cluster-sets, generated with different cluster methods (far left column). The entry clusters indicate how many clusters were generated by which method. Corpus size indicates how many reports remained after clustering, since several reports were identified as outliers and sorted out. **HDBSCAN! (HDBSCAN!)** has the best silhouette score as well as the best cls accuracy score. Although top2vec has an acceptable silhouette score, it is notable for its very poor predictability (cls accuracy: 0.372). Although k-means and **GSDPMM! (GSDPMM!)** have low silhouette scores, they are well predictable.

**Table 2:** Annotated topic words (translated from German to English), extracted from the **HDBSCAN! (HDBSCAN!)** cluster-set, using the tf–idf based extraction method. A particularly large number of topic words strongly refer to cluster names (left column) highlighted in green (strong cluster names). In the case of cluster names marked in orange, only a few topic words indicated the specified cluster name (weak cluster name). The same applies to the colour-coded topic words: topic words that strongly indicate a cluster name are highlighted in green (strong topic words). Orange highlighted topic words only weakly indicate a cluster name (weak topic words).

Cluster Index-Cluster Name	Keywords According to tf–idf
0-systemic lupus erythematosus	scale, chronicity_index, class, activity_index, nih, lupus_nephritis, iv, who, glomerular, iii
1-rapid progressive glomerulonephritis	quantity, glomeruli, scarred, necrosis, fresh, proliferating_glomerulonephritis, approx, segmental_necrotizing, extracapillary, concerning
2-tubulo-interstitial nephritis	approx, concerning, cortex, minor, immunostaining, damage, included, moderate, chronic, supplementary
3-pauci immune glomerulonephritis	of_this, glomeruli, total_amount, intact, extracapillary, scarred, global, necrosis, proliferating_glomerulonephritis, tubulointerstitial_damage
4-IgA nephritis	oxford, m1, e0, s1, t0, c0, classification, iga, glomerulonephritis, cell_count
5-FSGN	fsg, primary, distinction, focal, secondary, collapsing, chronic, glomerulosclerosis, take_place, hint
6-thrombotic microangiopathy	microangiopathy, thrombotic, active, reparation, preglomerular, glomerular, located, chronic, tubulointerstitial_damage, hypertension
7-multiple myeloma	nephropathy, kappa, cast, amyloidosis, myeloma_kidney, lcdd, a_notice, chronic, no, tubulointerstitial_damage
8-kidney transplant	rejection, routinely, chronic, success, immunohistochemical, examination, toxicity, hint, nephrosclerosis, mild
9-unremarkable finding	renal_parenchyma, unremarkable, left_over, mainly, normal, special, acute, tubular_damage, histological, glomerulonephritis
10	cut_level, hardly, noteworthy, chronic, tubulointerstitial_damage, deep, so_far, processing, nephrosclerosis, mild
11	microscopy, conventional, requirement, result, renal_parenchyma, foresee, chronic, nephrosclerosis, mild, examination
12 - membranous glomerulonephritis	pla2r, membranous, honorable, glomerulonephritis, stage, churg, chronic, tubulointerstitial_damage, positive, nephrosclerosis
13-diabetic glomerulosclerosis	diabetic_glomerulosclerosis, chronic, tubulointerstitial_damage, nodular, light_microscopic, picture, nephrosclerosis, difficult, examination, consist
14-glomerulosclerosis	glomerulosclerosis, global, tubulointerstitial_damage, chronic, nephrosclerosis, focal_segmental, focal, moderate, take_place, secondary
15-tubulo-interstitial nephritis	a_mild, tubular_damage, acute, chronic, nephrosclerosis, tubulointerstitial_damage, mild, a_moderate, moderate, potentially_reversible

**Table 3:** Annotated topic words (translated from German to English), extracted from the **HDBSCAN! (HDBSCAN!)** cluster-set, using the **SVM! (SVM!)** based extraction method. A particularly large number of topic words strongly refer to cluster names (left column) highlighted in green (strong cluster names). In the case of cluster names marked in orange, only a few topic words indicated the specified cluster name (weak cluster name). The same applies to the colour-coded topic words: topic words that strongly indicate a cluster name are highlighted in green (strong topic words). Orange highlighted topic words only weakly indicate a cluster name (weak topic words).

Cluster Index-Cluster Name	Keywords According to SVM
0-systemic lupus erythematosus	scale, chronicity_index, activity_index, class, -nih, iv, lupus_nephritis, component, iii, who
1-rapid progressive glomerulonephritis	quantity, sclerosing, glomeruli, scarred, glomerulus, fresh, of_which1, proliferating_glomerulonephritis, necrosis, sclerosed
2-tubulo-interstitial nephritis	tubular_epithelial_damage, moderate, minor, damage, none, completion, fibrosis, finally, known, tubulointerstitial
3-pauci immune glomerulonephritis	of_this, total_amount, intact, pauci_immune_glomerulonephritis, necrotizing, glomeruli, scarred, *, remaining, extracapillary
4-IgA nephritis	oxford_classification, e0, s1, m1, t0, c0, iga_glomerulonephritis, s0, applicable, e1
5-FSGN	fsg, primary, distinction, look_together, secondary, segmental_glomerulosclerosis, collapsing, at_most, continuing, patient
6-thrombotic microangiopathy	microangiopathy, thrombotic, reparation, preglomerular, glomerular, located, active, glomerular_thrombotic, hypertension, overwhelmingly
7-multiple myeloma	cast_nephropathy, myeloma_kidney, lcdd, lambda, kappa, amyloidosis, followed_by, light_chains, al-amyloidosis, light_chain_nephropathy
8-kidney transplant	rejection, routinely, success, examination, calcineurin_inhibitor_toxicity, ascending, humorous, bacterial, urinary_tract_infection, follow-up_report
9-unremarkable finding	renal_parenchyma, unremarkable, normal, largely, left_over, for_now, furthermore, special, iga-, pathological
10	hardly, cut_level, noteworthy, deep, so_far, processing, using, congo_red_coloring, to_exclusion, cellularor
11	microscopy, conventional, requirement, foresee, mild, membranous, early, cell_proliferation, result, g
12 - membranous glomerulonephritis	membranous, proteinuria, as_a_result, glomerulonephritis, pla2r, stage, churg, honorable, electron_microscopy, pla2r_positive
13-diabetic glomerulosclerosis	diabetic_glomerulosclerosis, consist, immune_complex_glomerulonephritis, nodular, picture, light_microscopic, partly, arteriolohyalinosis, diabetic_glomerulosclerosis, additionally
14-glomerulosclerosis	global, focal_segmental, segmental_glomerulosclerosis, glomerulosclerosis, focal_global, diffusesegmental, incl, focal, tubulointerstitial_damage, scarring
15-tubulo-interstitial nephritis	tubular_damage, a_mild, mild, tubulointerstitial_damage, change, a_moderate, mild, constantly, acute, malignancy

**Table 4:** Performance of different classification models, trained with the HDBSCAN cluster-set.

Classifier	F1-Score	Cohen’s Kappa Coefficient
Patho-BERT	0.667	0.631
SGD-classifier	0.644	0.598
MLP-classifier	0.639	0.599
German-BERT	0.610	0.572
Logistic Regression	0.589	0.567
CNN + embeddings	0.523	0.450
RNN + embeddings	0.464	0.394
Multinomial NB	0.442	0.370

F1-score and Cohen’s kappa coefficient of the tested classification methods, which were trained to predict the **HDBSCAN!** clustered data set. Each score is determined with ten-fold cross-validation. The transformer based model Patho-BERT and the **SVM! (SVM!)**-based SGD-classifier performed best.

**Table 5:** Classification performance of the Patho-BERT-classifier, predicting the HDBSCAN cluster-set.

Cluster/Diagnostic Group	F1-Score	Support
3	0.892	72
2	0.880	324
1	0.847	51
8	0.728	76
4	0.601	71
15	0.545	78
13	0.529	56
12	0.417	26
10	0.367	23
9	0.364	31
7	0.333	19
14	0.312	19
11	0.160	17
0	0.000	18
5	0.000	14
6	0.000	11

Cluster-Predictability of the **HDBSCAN!** cluster-set, using Patho-BERT as classifier. The cluster predictability was determined with the F1-score and the table is sorted by descending F1-scores. Each F1-score is the result of a 10 fold cross validation (average of 10 test measurements). Cluster 3 has the highest F1-score. Cluster 2 has a particularly strong support, which means this cluster is particularly large (324 documents) and was therefore often seen during training. The support specifies how many documents a cluster consists of. It can be observed that especially the smaller clusters could be recognized with difficulty or not at all.

## Data Availability

The code for this work is available on GitLab: http://gitlab.medma.uni-heidelberg.de/mlegnar/NLP-in-diagnostic-texts-from-nephropathology (14 July 2022). Furthermore, the vectorized or embedded text documents are available on HeiData: https://doi.org/10.11588/data/KS5W0H (14 July 2022). The raw texts (i.e., descriptive and diagnostic sections) are explicitly not made available, since it cannot be ruled out here that it is possible to infer the patient or the person making the report. This is in accordance with our local ethics committee.

## Abbreviations

The following abbreviations are used in this manuscript:

BoW	Bag-of-Words
SVM	support vector machine
NLP	natural language processing
OOV	out of vocabulary
MLM	masked language modeling
LSTM	long short-term memory
ICD-O	International Classification of Diseases for Oncology
CNN	convolutional neural network
RNN	Recurrent Neural Network
BERT	Bidirectional Encoder Representations from Transformers
UMAP	Uniform Manifold Approximation and Projection
HDBSCAN	Hierarchical Density-Based Spatial Clustering of Applications with Noise
GSDPMM	Gibbs Sampling algorithm for the Dirichlet Process Multinomial Mixture
SGD	Stochastic Gradient Descent
PCA	Principal Component Analysis
tf-idf	term frequency–inverse document frequency
NMI	normalized mutual information
ReLU	rectified linear unit
VL-PTMs	Vision-Language Pre-Trained Models
s-score	silhouette score
cls accuracy	classification accuracy
ML	machine learning
LDA	Latent Dirichlet Allocation
NB	naive bayes
MLP	multilayer perceptron
UMLS	Unified Medical Language System
SMOTE	synthetic minority over-sampling technique

## Appendix A. Additional Annotated Topic Word Tables

**Table A1:** Annotated German topic words, extracted from the HDBSCAN! (**HDBSCAN!**) cluster-set, using the tf–idf based extraction method. A particularly large number of topic words strongly refer to cluster names (left column) highlighted in green (strong cluster names). In the case of cluster names marked in orange, only a few topic words indicated the specified cluster name (weak cluster name). The same applies to the colour-coded topic words: topic words that strongly indicate a cluster name are highlighted in green (strong topic words). Orange highlighted topic words only weakly indicate a cluster name (weak topic words).

Cluster Index-Cluster Name	Keywords according to tf-idf
0-Systemischer Lupus erythematodes	skala, chronizitätsindex, klasse, aktivitätsindex, nih, lupus_nephritis, iv, who, glomerulärer, iii
1-Rapid progressive Glomerulonephritis	anzahl, glomeruli, vernarbten, nekrosen, frisch, proliferierende_glomerulonephritis, ca, segmental_nekrotisierend, extrakapillär, betreffend
2-Tubulo-interstitielle Nephritis	ca, betreffend, cortex, leicht, immunfärbung, schädigung, miterfasst, mäßig, chronisch, ergänzend
3-Pauci-Immun-Glomerulonephritis	hiervon, glomeruli, gesamtzahl, intakt, extrakapillär, vernarbt, global, nekrosen, proliferierende_glomerulonephritis, tubulointerstitieller_schaden
4-Ig A Nephritis	oxford, m1, e0, s1, t0, c0, klassifikation, iga, glomerulonephritis, zellzahl
5-FSGN	fsg, primär, unterscheidung, fokal, sekundär, kollabierend, chronisch, glomerulosklerose, erfolgen, hinweis
6-Thrombotic microangiopathy	mikroangiopathie, thrombotisch, floride, reparation, präglomerulär, glomeruläre, befindlich, chronisch, tubulointerstitieller_schaden, hypertonie
7-Multiples Myelom	nephropathie, kappa, cast, amyloidose, myelomniere, lcdd, hinweis, chronisch, kein, tubulointerstitieller_schaden
8-Nierentransplantat	abstoßung, routinemäßig, chronisch, erfolg, immunhistochemisch, untersuchung, toxizität, hinweis, nephrosklerose, leichtgradig
9-Unauffälliger Befund	nierenparenchym, unauffällig, übrig, weitgehend, normal, speziell, akut, tubulusschaden, histologisch, glomerulonephritis
10	schnittstufe, kaum, nennenswert, chronisch, tubulointerstitieller_schaden, tief, bislang, aufarbeitung, nephrosklerose, leichtgradig
11	mikroskopie, konventionell, maßgabe, ergeben, nierenparenchym, absehen, chronisch, nephrosklerose, leichtgradigen, untersuchung
12- Membranöse Glomerulonephritis	pla2r, membranöse, ehrenreich, glomerulonephritis, stadium, churg, chronisch, tubulointerstitieller_schaden, positiv, nephrosklerose
13- Diabetische Glomerulosklerose	diabetische_glomerulosklerose, chronisch, tubulointerstitieller_schaden, nodulär, lichtmikroskopisch, bild, nephrosklerose, schwer, untersuchung, bestehen
14-Glomerulosklerose	glomerulosklerose, global, tubulointerstitieller_schaden, chronisch, nephrosklerose, fokal_segmental, fokal, mäßiggradig, erfolgen, sekundär
15-Tubulo-interstitielle Nephritis	leichtgradiger, tubulusschaden, akut, chronisch, nephrosklerose, tubulointerstitieller_schaden, leichtgradig, mäßiggradiger, mäßiggradig, potentiell_reversibel

**Table A2:** Annotated German topic words, extracted from the **HDBSCAN**! (**HDBSCAN!**) cluster-set, using the **SVM!** (**SVM!**) based extraction method. A particularly large number of topic words strongly refer to cluster names (left column) highlighted in green (strong cluster names). In the case of cluster names marked in orange, only a few topic words indicated the specified cluster name (weak cluster name). The same applies to the colour-coded topic words: topic words that strongly indicate a cluster name are highlighted in green (strong topic words). Orange highlighted topic words only weakly indicate a cluster name (weak topic words).

Cluster Index-Cluster Name	Keywords according to SVM
0-Systemischer Lupus erythematodes	skala, chronizitätsindex, aktivitätsindex, klasse, -nih, iv, lupus_nephritis, komponente, iii, who
1-Rapid progressive Glomerulonephritis	anzahl, sklerosieren, glomeruli, vernarbten, glomerulus, frisch, davon1, proliferierende_glomerulonephritis, nekrosen, sklerosierten
2-Tubulo-interstitielle Nephritis	tubulusepithelschaden, mäßig, leicht, schädigung, keine, komplettierung, fibrose, abschließend, bekannt, tubulointerstitiell
3-Pauci-Immun-Glomerulonephritis	hiervon, gesamtzahl, intakt, pauci-immun-glomerulonephritis, nekrotisierend, glomeruli, vernarbt, *, restlich, extrakapillär
4-Ig A Nephritis	oxford-klassifikation, e0, s1, m1, t0, c0, iga-glomerulonephritis, s0, anwendbar, e1
5-FSGN	fsg, primär, unterscheidung, zusammenschau, sekundär, segmentale_glomerulosklerose, kollabierend, allenfalls, weiterführend, patient
6-Thrombotic microangiopathy	mikroangiopathie, thrombotisch, reparation, präglomerulär, glomeruläre, befindlich, floride, glomerulärethrombotisch, hypertonie, überwiegend
7-Multiples Myelom	cast-nephropathie, myelomniere, lcdd, lambda, kappa, amyloidose, anschließen, leichtketten, al-amyloidose, leichtkettennephropathie
8-Nierentransplantat	abstoßung, routinemäßig, erfolg, untersuchung, calcineurininhibitor-toxizität, aufsteigend, humorale, bakteriell, harnwegsinfekt, nachbericht
9-Unauffälliger Befund	nierenparenchym, unauffällig, normal, weitgehend, übrig, vorbehaltlich, imübrigen, speziell, iga-, pathologisch
10	kaum, schnittstufe, nennenswert, tief, bislang, aufarbeitung, mittels, kongorot-färbung, zumausschluss, zelluläreoder
11	mikroskopie, konventionell, maßgabe, absehen, leichtgradigen, membranösen, früh, zellvermehrung, ergeben, g
12-Membranöse Glomerulonephritis	membranöse, proteinurie, infolge, glomerulonephritis, pla2r, stadium, churg, ehrenreich, elektronenmikroskopie, pla2r-positiv
13-Diabetische Glomerulosklerose	diabetische_glomerulosklerose, bestehen, immunkomplexglomerulonephritis, nodulär, bild, lichtmikroskopisch, teils, arteriolohyalinose, diabetische_glomeruloskleros, zusätzlich
14-Glomerulosklerose	global, fokal_segmental, segmentaleglomerulosklerose, glomerulosklerose, fokalglobale, diffussegmental, einschl, fokal, tubulointerstitieller_schaden, vernarbung
15-Tubulo-interstitielle Nephritis	tubulusschaden, leichtgradiger, leichtgradig, tubulointerstitieller_schaden, veränderung, mäßiggradiger, leichtgradige, andauernd, akut, malignität
